# Alvarenga Prize of the College of Physicians of Philadelphia

**Published:** 1892-03

**Authors:** Charles W. Dulles


### Alvarenga Prize of the College of Physicians of Philadelphia.

The College of Physicians of Philadelphia announces that the next award of the Alvarenga Prize, being the income for one year of the bequest of the late Senor Alvarenga, and amounting to about One Hundred and Eighty Dollars, will be made on July 14th, 1892. Essays intended for competition may be upon any subject in Medicine, and must be received by the Secretary of the College on or before May 1st, 1892. It is a condition of competition that the successful essay or a copy of it shall remain in possession of the College. CHARLES W. DULLES, *Secretary*.

